# Dacomitinib in Combination with chemotherapy is effective in lung adenocarcinoma with rare *EGFR* L747P mutation and bone metastases: a case report

**DOI:** 10.3389/fonc.2026.1762855

**Published:** 2026-05-05

**Authors:** Weixin Su, Haiyan Deng, Zhi Yang, Anjian Gao, Bo Gao

**Affiliations:** Department of Respiratory Medicine, Shenzhen Second People’s Hospital, Shenzhen, Guangdong, China

**Keywords:** case report, dacomitinib, EGFR exon 19, EGFR L747P, lung adenocarcinoma

## Abstract

**Background:**

Rare epidermal growth factor receptor (*EGFR*) mutations have a low incidence, and their response to *EGFR* tyrosine kinase inhibitors (TKI) has not been sufficiently studied. L747P is a rare *EGFR* mutation located in exon 19. Previous case reports have demonstrated the efficacy of dacomitinib for *EGFR* L747P mutation. This case report specifically investigates the therapeutic effects of dacomitinib in combination with chemotherapy for L747P mutation and bone metastases, with longer efficacy observation and deeper exploration of molecular mechanisms.

**Case Description:**

We present the case of a 56-year-old female with lung adenocarcinoma and bone metastases. Next-generation sequencing (NGS) detected an *EGFR* L747P mutation. The patient initially received pemetrexed plus carboplatin chemotherapy. However, after three cycles, tumor markers continued to rise, pulmonary lesions showed insignificant regression, and bone metastases progressed. Dacomitinib was subsequently added to the treatment regimen. Tumor markers decreased one month after initiating combined therapy, and follow-up imaging at 2 and 6 months revealed regression of both pulmonary lesions and bone metastases. The patient achieved a progression-free survival (PFS) of 16 months. The treatment efficacy was evaluated as a partial response (PR), with manageable adverse events including skin rash and paronychia.

**Conclusion:**

Dacomitinib may provide sustained clinical benefit and an acceptable safety profile in lung adenocarcinoma patients with L747P mutation and bone metastases.

## Introduction

Lung cancer remains the leading cause of cancer-related mortality worldwide (1). Non-small cell lung cancer (NSCLC) represents the most prevalent subtype, accounting for approximately 85% of all cases; consequently, reducing the morbidity and mortality of NSCLC is crucial for overall lung cancer control (2, 3). Lung adenocarcinoma (LUAD) constitutes the majority of NSCLC subtypes (4).

In LUAD, epidermal growth factor receptor (*EGFR*) mutations are detected in approximately 15% of cases (5). Approximately 38% of Chinese patients with NSCLC exhibit a mutation in the EGFR gene (6). Exon 19 deletions and the exon 21 L858R mutation are the most common actionable genomic alterations in NSCLC patients (7). Among these, deletions within exon 19 account for about 45% of all *EGFR* mutations in NSCLC (8). *EGFR* mutations other than 19Del and L858R are called rare mutations and account for ~10% of all EGFR mutations, which represents a highly heterogeneous group with 600 variants identified (9). The p.L747P missense mutation, occurring in exon 19 of the *EGFR* gene, is rare in NSCLC. Common *EGFR* mutations are sensitive to *EGFR*-tyrosine kinase inhibitors (TKIs) (10). However, due to the low incidence of rare *EGFR* mutations, their responses to *EGFR*-TKIs have not been sufficiently investigated. Recent studies show that although first-generation *EGFR*-TKIs exhibit limited efficacy in patients with rare EGFR mutations, afatinib, a second-generation *EGFR*-TKI, demonstrates improved clinical activity in this population, with a response rate (RR) of 71.1% and progression-free survival (PFS) of 10.7 months (11). Osimertinib, a third-generation *EGFR*-TKI, appears limited for rare *EGFR* mutations, with a reported RR of 50% and PFS of 8.2 months (12, 13). Additionally, the *EGFR* rare mutation form of LUAD shows a higher immune activation state, indicating it as a potential target for PD-1/PD-L1 inhibitor therapy (14).

While previous case reports have indicated that potential efficacy of dacomitinib in *EGFR* L747P mutation (15), clinical evidence specifically addressing the efficacy of dacomitinib in *EGFR* L747P mutation with bone metastases remains limited, and the underlying molecular mechanisms have yet to be fully elucidated.In this context, we report a case of LUAD with bone metastases. Next-generation sequencing (NGS), which aids in the molecular subtyping of NSCLC patients (16), identified a rare *EGFR* L747P mutation in this patient. The patient achieved a partial response (PR) following treatment with dacomitinib in combination with chemotherapy, with PFS of 16 months. Subsequently, isolated pelvic progression developed after 16 months, suggesting possible clonal evolution.

## Case presentation

**Figure 1:**
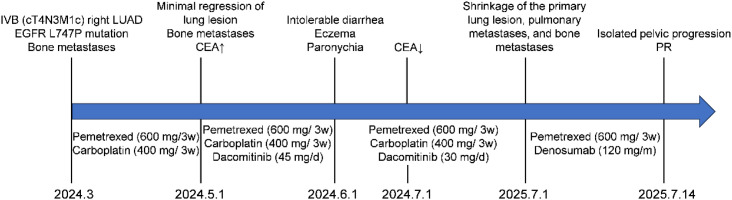
Key events during the patient’s clinical course.

**Figure 2:**
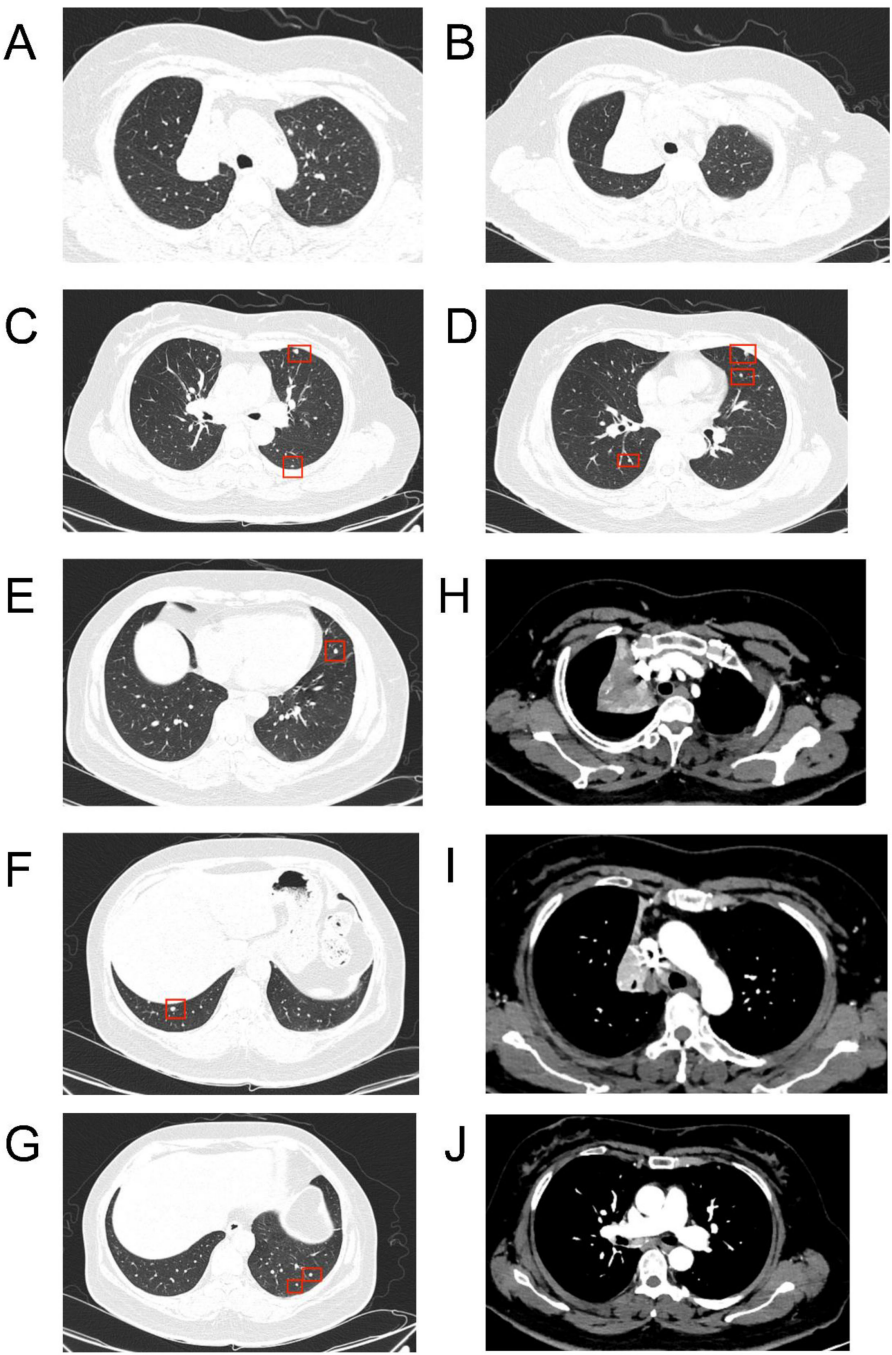
CT images at the patient’s initial visit. **(A)** Complete atelectasis of the right upper lobe; **(B)** Obstruction of the right upper lobe bronchus; **(C)** Multiple solid nodules in the left upper lobe; **(D–G)** Multiple solid nodules in both lungs; **(H)** Mass-like soft tissue density shadow in the right hilar region, showing marked heterogeneous enhancement with ill-defined borders on contrast-enhanced imaging; **(I)** Multiple small lymph nodes visible within the mediastinum; **(J)** Multiple small mediastinal lymph nodes are visible.

**Figure 3:**
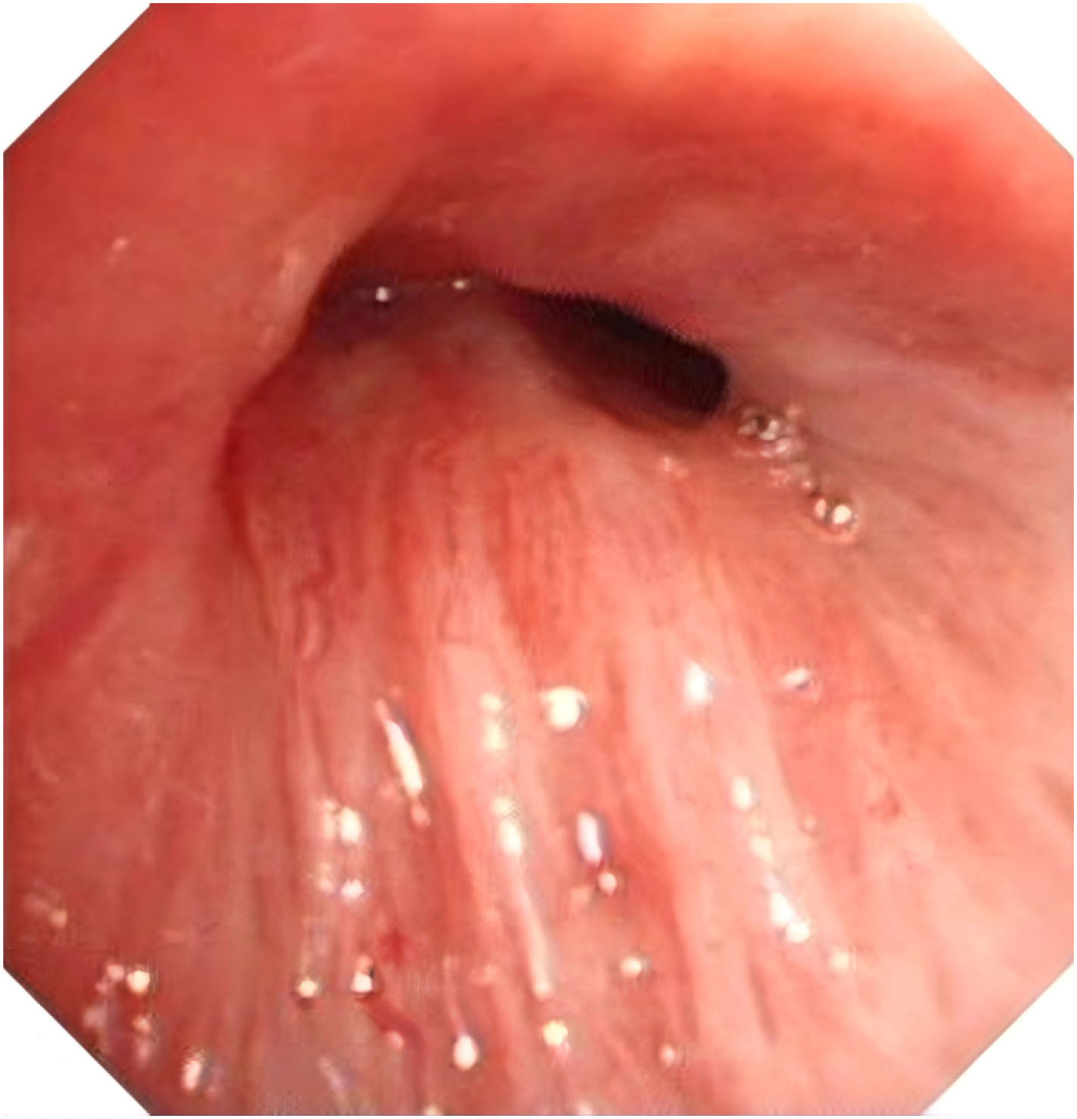
Patient bronchoscopy results.

**Figure 4:**
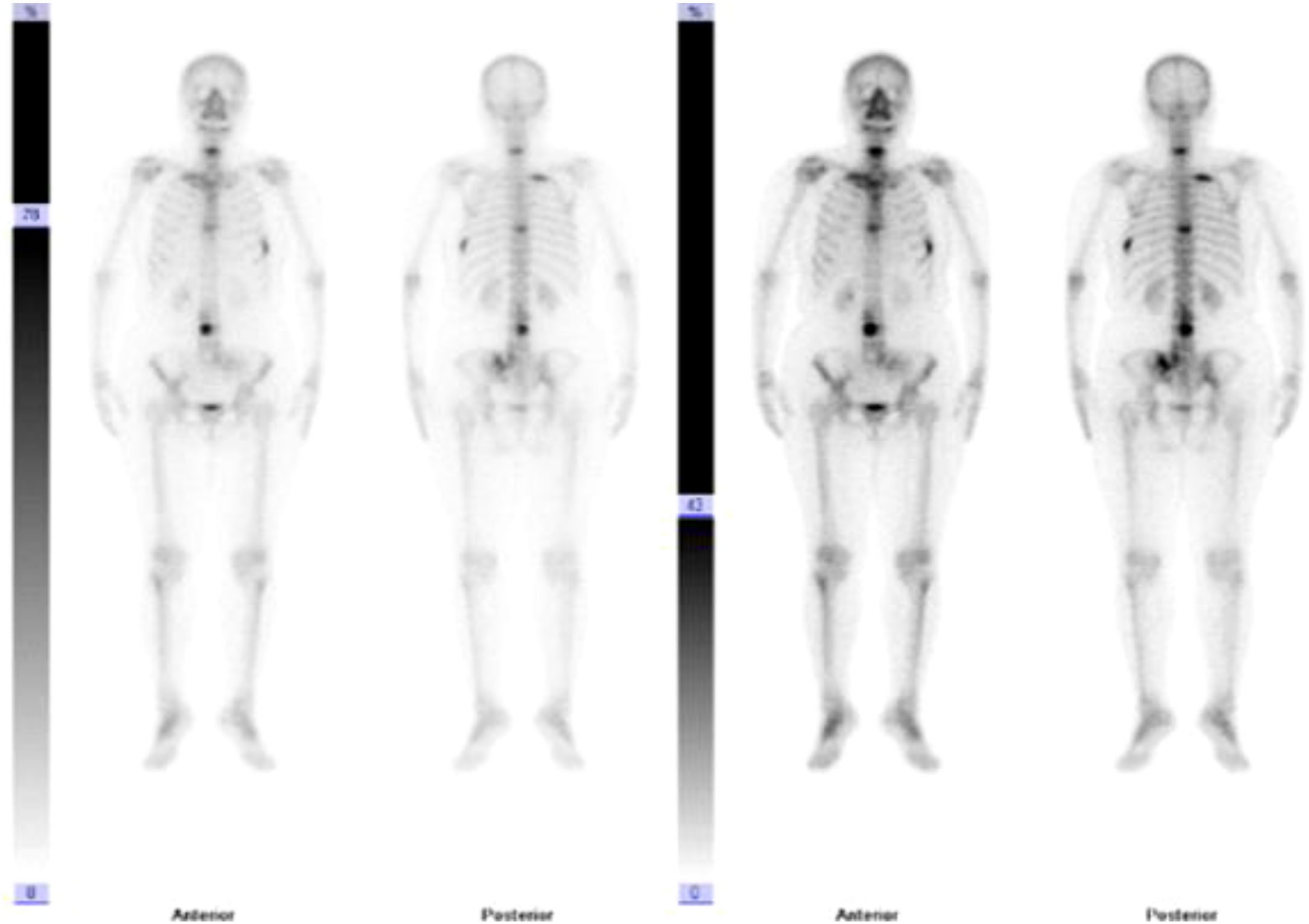
Bone scan results at the patient’s initial consultation.

**Figure 5:**
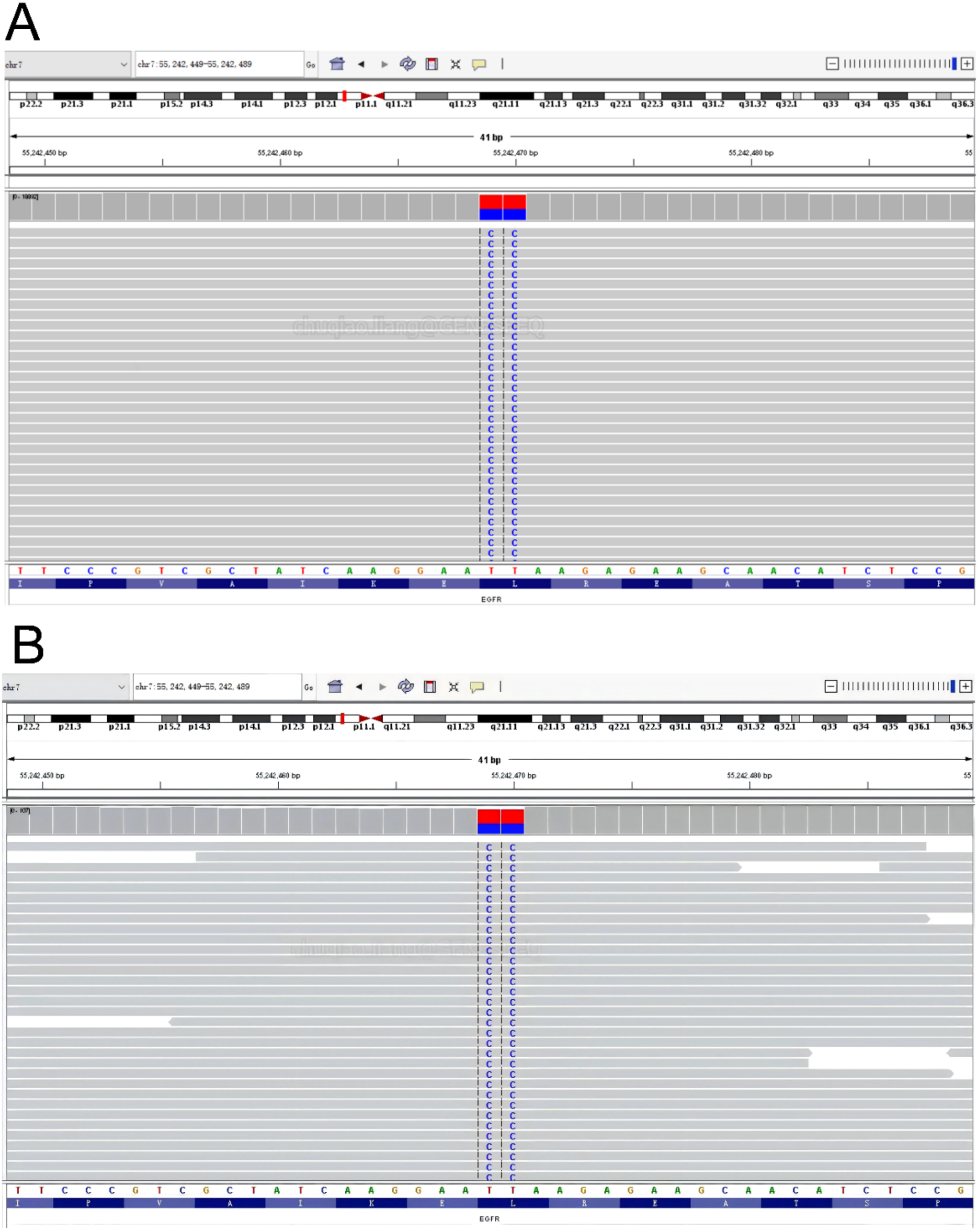
NGS results of patient plasma samples and tumor tissue performed on the Illumina NovaSeq 6000 platform using a targeted 139-gene LungTrak panel (Geneseeq, China). Results from **(A)** lung tumor tissue samples and **(B)** peripheral blood demonstrate the presence of the EGFR Exon19 c.2239_2240delinsCC p.L747P mutation. The reference sequence for the EGFR gene is shown as the baseline for DNA sequences.

In March 2024, a 56-year-old Chinese woman with no smoking history was found to have atelectasis in the right upper lobe by chest computed tomography (CT) scan. The patient subsequently developed intermittent dull pain in the right anterior chest and discomfort in the right neck, leading to hospitalization in the Department of Respiratory Medicine (**Figure 1**). Enhanced CT of the chest revealed complete atelectasis of the right upper lobe with a poorly defined soft tissue mass in the right hilum, inseparable from the collapsed lung. The mass showed heterogeneous enhancement after contrast administration and occluded the right upper lobe bronchus with indistinct margins. Scattered patchy and linear opacities were observed in both lungs, along with multiple solid nodules. The largest nodule (11 mm × 6 mm) was located in the superior lingular segment of the left upper lobe and was well-defined. Multiple small lymph nodes were visible. Lytic lesions were identified in the T8, T9, and L2–L5 vertebrae and their appendages, suspicious for metastases (**Figure 2**). Bronchoscopy revealed stenosis at the orifice of the right upper lobe (**Figure 3**). Malignant cells were detected in bronchoalveolar lavage fluid; subsequent right supraclavicular lymph node biopsy confirmed metastatic moderately differentiated LUAD. Immunohistochemistry (IHC) results were as follows: TTF-1 (+), CK7 (+), P40 (-), ALK (D5F3) (-), ALK-Neg (-), Her-2 (0). The whole-body bone scintigraphy confirmed focal abnormal tracer uptake in multiple sites, including the right 2nd posterior rib, left 7th rib (axillary segment), C6, T7, L2, L3, L5 vertebrae, and the left ilium, consistent with bone metastases (**Figure 4**). The final diagnosis was stage IVB (cT4N3M1c) right lung adenocarcinoma with right supraclavicular lymph node and multiple bone metastases. We performed NGS using a panel covering 139 lung cancer-related genes on tumor tissue and plasma samples. The NGS results identified an *EGFR* exon 19 L747P mutation (*EGFR* Exon19 c.2239_2240delinsCC p.L747P) (**Figure 5**) with a mutant allele frequency of 41.67% and PD-L1 expression was negative (Tumor Proportion Score (TPS) <1%). Due to financial constraints, the patient initially declined *EGFR*-TKI targeted therapy. She was instead started on chemotherapy with pemetrexed and carboplatin.

**Figure 6:**
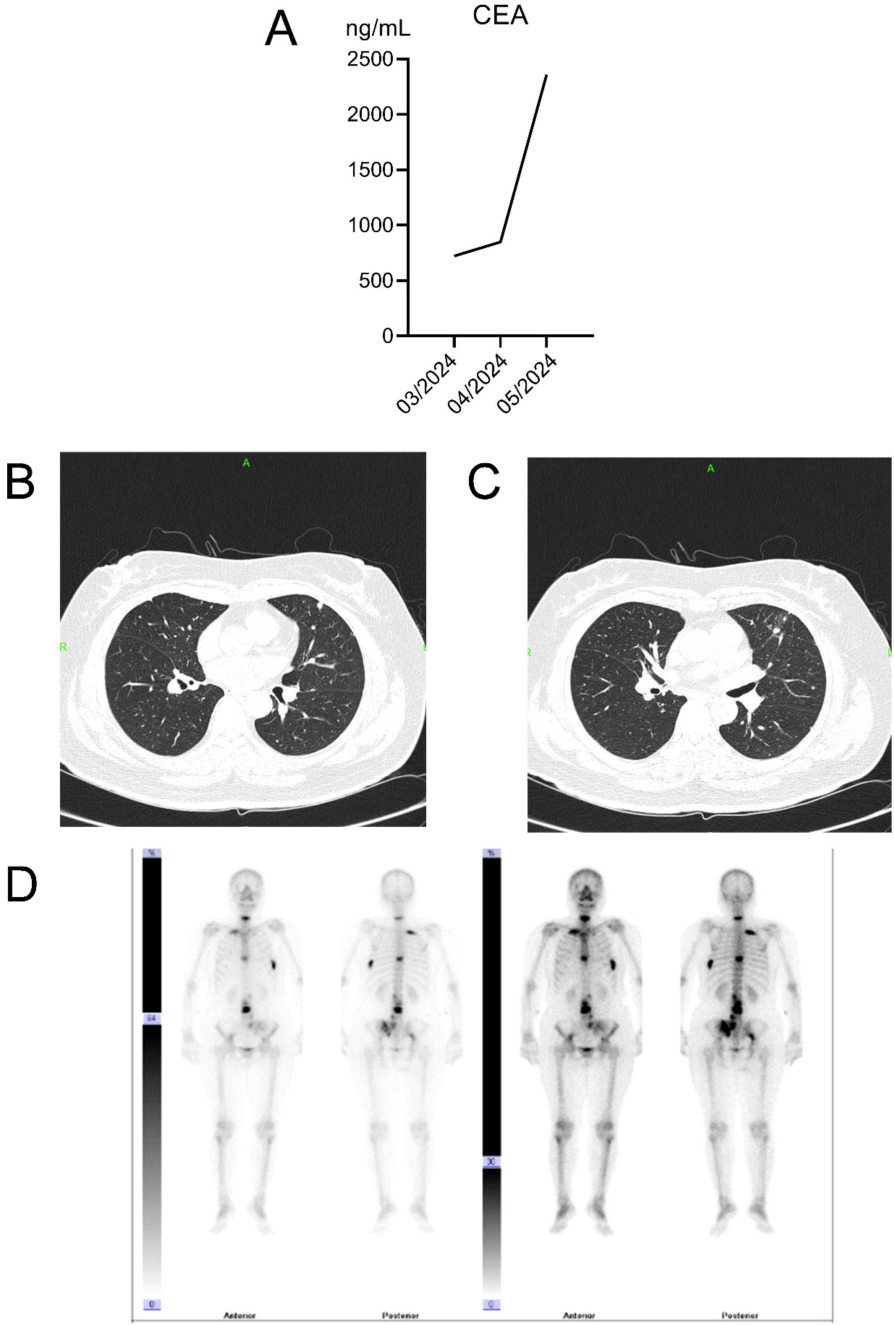
Changes in tumor and bone metastases following initial chemotherapy. **(A)** Trend of CEA levels; **(B)** Multiple solid nodules in both lungs prior to chemotherapy; **(C)** Increased number and enlarged size of multiple solid nodules in both lungs following chemotherapy; **(D)** Bone scan results after two cycles of chemotherapy.

**Figure 7:**
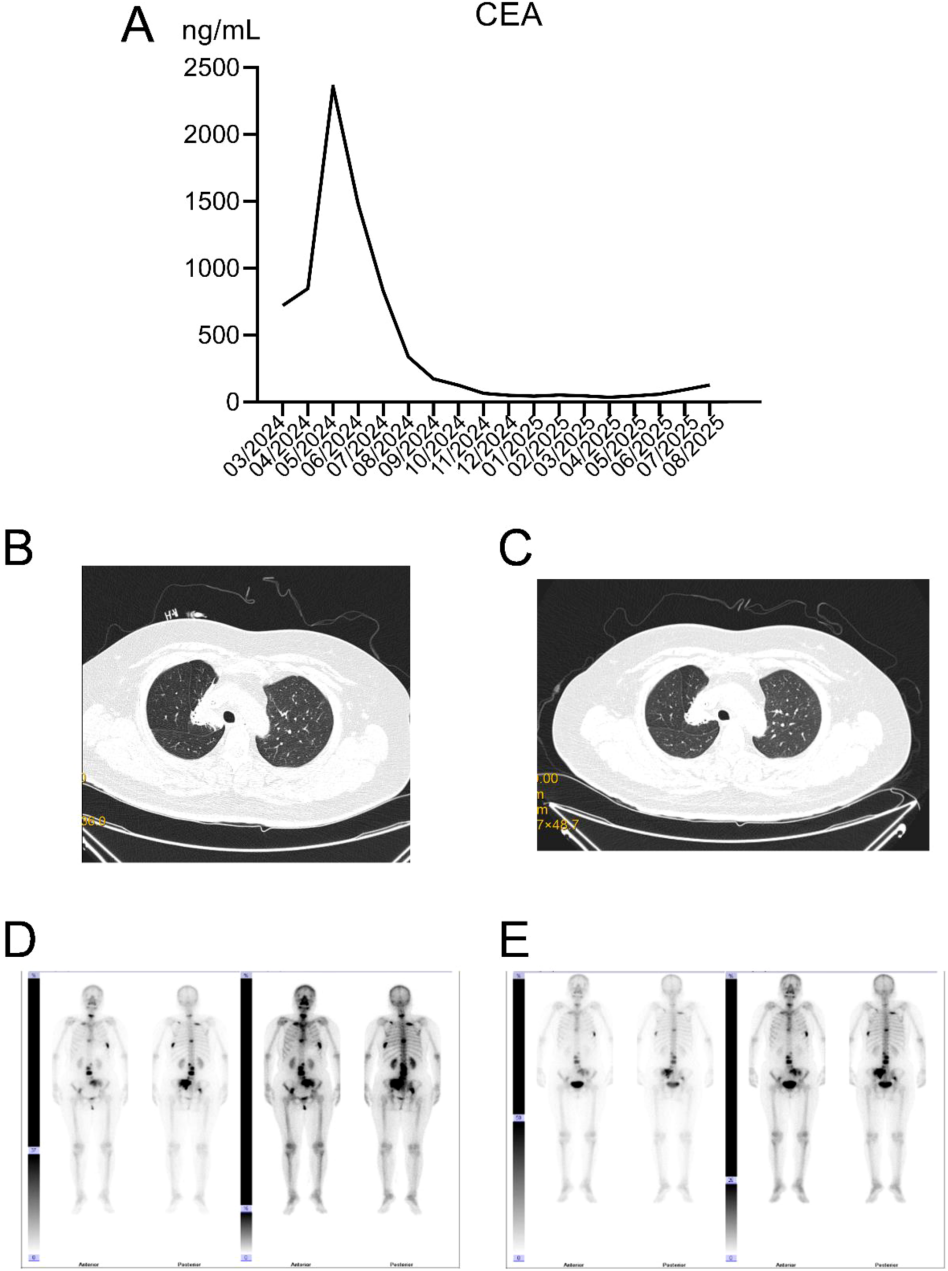
Changes in tumor and bone metastases following treatment with dacarbazine combined with chemotherapy. **(A)** Trend of CEA levels; **(B)** Reduction of the right upper lung target lesion after 7 months of targeted therapy combined with chemotherapy; **(C)** Further reduction of the right upper lung lesion after 15 months of targeted therapy and two courses of chemotherapy; **(D)** Bone scan showing the most severe bone metastases; **(E)** Bone scan after 6 months of combined therapy.

**Figure 8:**
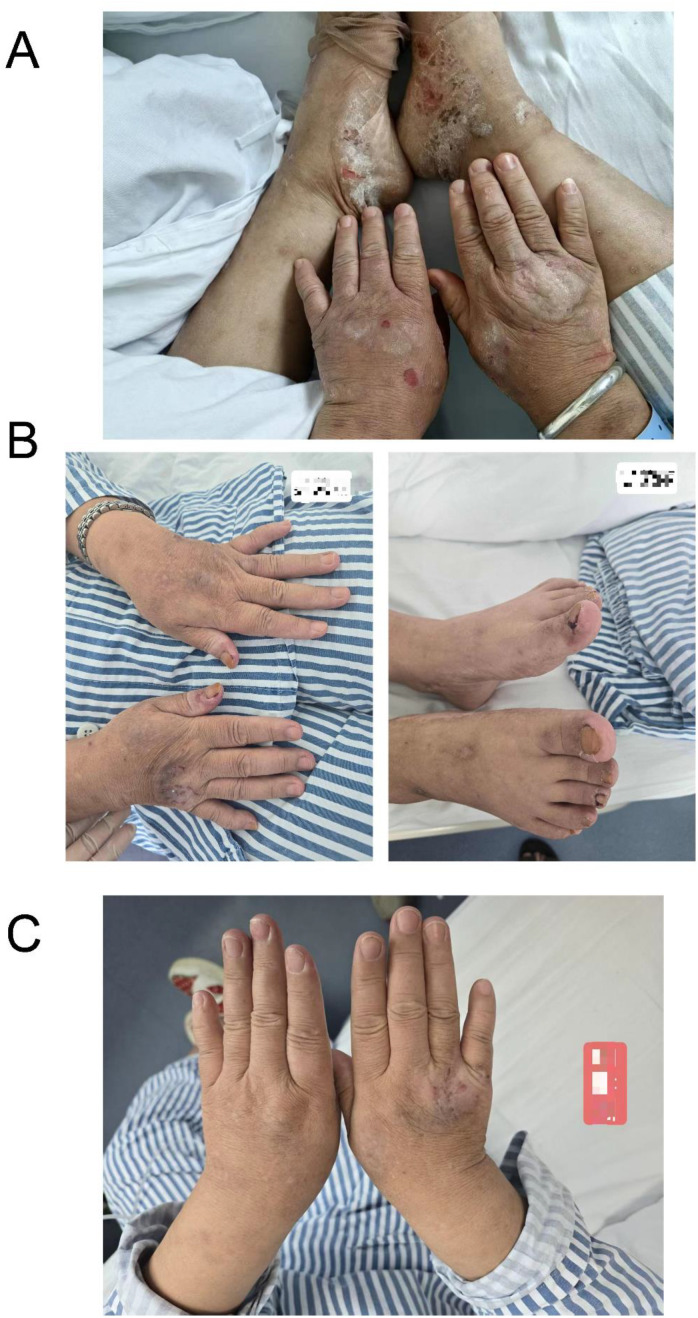
Images of hand and foot skin from the patient. **(A)** At initial consultation, **(B)** at the start of dacarbazine treatment, and **(C)** recent hand and foot skin images.

After three cycles of chemotherapy, the tumor marker carcinoembryonic antigen (CEA) continued to rise (**Figure 6A**). The right upper lung lesion showed insignificant regression, multiple small nodules in both lungs increased in number and size (**Figures 6B, C**), and bone metastases showed progression on scintigraphy (**Figure 6D**). Consequently, on May 1, 2024, the patient began oral dacomitinib alongside chemotherapy. Due to intolerable diarrhea, eczema, and paronychia, the dacomitinib dose was reduced after one month. One month after initiating combined dacomitinib therapy, the patient’s CEA levels began to decline steadily (**Figure 7A**). Follow-up contrast-enhanced CT and bone scans at 2 and 6 months showed continuous shrinkage of the primary lung lesion, pulmonary metastases, and bone metastases, with pulmonary metastases nearly disappearing (**Figures 7B–E**). The patient continued dacomitinib treatment and completed a total of 6 cycles of pemetrexed plus carboplatin chemotherapy, followed by maintenance therapy with pemetrexed alone. Denosumab was administered for bone health. Isolated progression in a left pelvic bone lesion was noted on July 14, 2025, while lung lesions continued to shrink. The progression-free survival (PFS) was 16 months. During treatment, the patient experienced manageable skin eczema and paronychia (**Figures 8A, B**), which improved with topical medication (**Figure 8C**), with no severe adverse events reported.

## Discussion

Compared to common *EGFR* mutations, rare *EGFR* mutations exhibit heterogeneous molecular characteristics, distinct epidemiological features, varied responses to *EGFR*-TKI therapy, and are often associated with shorter PFS (17). NGS is routinely used for lung cancer genomic profiling to identify known, actionable, non-synonymous driver mutations, which has identified numerous oncogenic driver mutations, including *EGFR* (18, 19), tumor protein p53 (*TP53*) (20), kirsten rat sarcoma viral oncogene (*KRAS*) (21), RB transcriptional corepressor 1 (*RB1*) (22), MET pro-to-oncogene (*MET*) (1). These genomic alterations significantly influence disease progression, therapeutic resistance, and clinical outcomes. *TP53* co-mutations were the most common types, especially *TP53*-*EGFR* and *TP53*-*KRAS* co-mutations (23). Such co-mutations contribute to intra-tumor molecular heterogeneity and can drive clonal evolution of the cancer (24–26). Importantly, the presence of co-alterations has been linked to variability in clinical behavior—for instance, differences in growth rate, metastatic pattern, and drug sensitivity within what was thought to be a single molecular subtype of NSCLC (27).

The p.L747P missense mutation is caused by a two-base-pair substitution (c.2239_2240TT>CC) at codon 747, leading to a leucine-to-proline substitution that drives tumorigenesis (28). Preclinical studies have indicated that the L747P mutation confers reduced sensitivity to first-generation (e.g., erlotinib) and third-generation (e.g., osimertinib) *EGFR*-TKIs, while remaining sensitive to the second-generation TKI afatinib (29). In a single-arm phase II clinical trial, dacomitinib demonstrated significant efficacy and a favorable safety profile in advanced NSCLC patients with sensitizing uncommon EGFR mutations (30). However, due to the complexity and diversity of these mutations, current clinical practice lacks standardized, mutation-specific TKI treatment strategies. Optimal management strategies for patients harboring *EGFR* L747P mutation with bone metastasis remain poorly characterized. This case report highlights the marked efficacy of dacomitinib-chemotherapy combination therapy in controlling both the primary tumor and bone metastases, with long-term follow-up demonstrating a PFS duration of 16 months and no significant adverse events observed, indicating a favorable safety profile. Our finding highlights the importance of biopsy and molecular analysis in guiding treatment decisions, contributing to differential therapeutic responses and the development of resistance, emphasizing the necessity for personalized therapeutic approaches.

Kinetic simulations suggest that the L747P mutation significantly reduces the van der Waals interactions between the *EGFR* tyrosine kinase and first-generation TKIs like gefitinib, leading to insensitivity. Furthermore, the L747P mutation induces a structural change in the αC-helix orientation towards the P-loop, facilitating the formation of a salt bridge between K745 and E762 residues, which stabilizes the active conformation of *EGFR* (31). This mechanism explains the failure of initial chemotherapy and the potential inefficacy of first-generation TKIs in our patient. Dacomitinib readily crosses the blood-brain barrier (32) and accumulates in bone tissue (18), which may explain the 16-month control of bone metastases observed in this case. Furthermore, even during subsequent treatment when the dacomitinib dose was reduced from 45 mg daily to 30 mg daily, the patient maintained therapeutic efficacy.

This case, combined with previous literature and case reports, supports categorizing L747P as a “pan-second-generation TKI-sensitive mutation.” While current NCCN guidelines do not specify a treatment strategy for this mutation, we suggest prioritizing second-generation TKIs over chemotherapy or first-generation TKIs for such patients (33). Studies have shown that the second-generation *EGFR*-TKI afatinib extends PFS to 11.1 months, compared to 6.9 months with cisplatin-based chemotherapy (34). Our patient, treated with dacomitinib combined with pemetrexed and carboplatin, achieved a PFS of 16 months, suggesting the promise of an *EGFR*-TKI plus chemotherapy regimen. The isolated pelvic progression after 16 months in this case may indicate clonal evolution.

This study has inherent limitations as a single-case report, which restricts statistical generalizability. However, the clinical value of this case lies in the patient’s distinctive clinical features of *EGFR* L747P mutation with extensive bone metastases, and the remarkably prolonged PFS of 16 months achieved with dacomitinib-based therapy. Future prospective cohort studies should enroll additional patients with EGFR L747P mutation and bone metastases to further validate the durability and clinical utility of dacomitinib-containing combination regimens. Factors such as tumor heterogeneity, undetected co-mutations, or the immunomodulatory effects of chemotherapy may also warrant further investigation in future studies.

## Conclusion

This case demonstrates that dacomitinib combined with chemotherapy can significantly control disease progression in lung adenocarcinoma harboring the L747P mutation with bone metastases. This finding highlights the need for individualized TKI selection for rare *EGFR* mutations. Dacomitinib’s potent irreversible inhibition, favorable bone tissue distribution, and synergistic potential with chemotherapy provides a new therapeutic strategy for these patients.

## Data Availability

The datasets presented in this study can be found in online repositories. The names of the repository/repositories and accession number(s) can be found in the article/supplementary material.
